# Integration of GWAS, linkage analysis and transcriptome analysis to reveal the genetic basis of flowering time-related traits in maize

**DOI:** 10.3389/fpls.2023.1145327

**Published:** 2023-03-22

**Authors:** Xun Wu, Ying Liu, Xuefeng Lu, Liang Tu, Yuan Gao, Dong Wang, Shuang Guo, Yifei Xiao, Pingfang Xiao, Xiangyang Guo, Angui Wang, Pengfei Liu, Yunfang Zhu, Lin Chen, Zehui Chen

**Affiliations:** ^1^ Institute of Upland Food Crops, Guizhou Academy of Agricultural Sciences, Guiyang, Guizhou, China; ^2^ College of Agriculture, Guizhou University, Guiyang, Guizhou, China; ^3^ Institute of Animal Science, Chinese Academy of Agricultural Sciences, Beijing, China

**Keywords:** maize, genome-wide association study, flowering time, RNA-Seq, candidate gene

## Abstract

Maize (*Zea mays*) inbred lines vary greatly in flowering time, but the genetic basis of this variation is unknown. In this study, three maize flowering-related traits (DTT, days to tasselling; DTP, days to pollen shed; DTS, days to silking) were evaluated with an association panel consisting of 226 maize inbred lines and an F_2:3_ population with 120 offspring from a cross between the T32 and Qi319 lines in different environments. A total of 82 significant single nucleotide polymorphisms (SNPs) and 117 candidate genes were identified by genome-wide association analysis. Twenty-one quantitative trait loci (QTLs) and 65 candidate genes were found for maize flowering time by linkage analysis with the constructed high-density genetic map. Transcriptome analysis was performed for Qi319, which is an early-maturing inbred line, and T32, which is a late-maturing inbred line, in two different environments. Compared with T32, Qi319 showed upregulation of 3815 genes and downregulation of 3906 genes. By integrating a genome-wide association study (GWAS), linkage analysis and transcriptome analysis, 25 important candidate genes for maize flowering time were identified. Together, our results provide an important resource and a foundation for an enhanced understanding of flowering time in maize.

## 1 Introduction

Maize, one of the most important crops in the world (Yang and Yan, 2021), originated in the lowland tropics of South America. Since it was first domesticated 9000 years ago, its planting area has expanded (Matsuoka et al., 2002), and this spread to various parts of the globe has resulted in rich phenotypic and genetic variation (Li et al., 2016). Among the phenotypic variations in maize, the variation in flowering time is an important factor in determining adaptation to local environments and is also a key selection standard for maize breeding and germplasm innovation (Shi et al., 2022).

In recent decades, the main genetic factors controlling flowering time in the model plant *Arabidopsis thaliana* have begun to be determined (Song et al., 2013; Johansson and Staiger, 2015; Freytes et al., 2021). Moreover, some important flowering time genes from other plant species have been cloned, such as *FLOWERING LOCUS T* (*FT*), *CONSTANS* (*CO*), and *SUPPRESSOR OF OVEREXPRESSION OF CONSTANS* (*SOC1*), which has been helpful for understanding the plant molecular regulatory network controlling flowering time (Wickland and Hanzawa, 2015; Liu et al., 2008; Shim et al., 2017). In maize, flowering time shows rich variation, with the earliest flowering time being 35 days and the latest being 120 days (Colasanti and Muszynski, 2009). Recently, many genes that regulate maize flowering time have been cloned (Kozaki et al., 2004; Hung et al., 2012; Yang et al., 2013; Guo et al., 2018). For example, *ZmCCT* is a homologous gene of the rice photoperiod response regulator *Ghd7* (Hung et al., 2012; Yang et al., 2013), *ZCN8* is homologous to the Arabidopsis *FT* gene (Guo et al., 2018), and *ID1* has a zinc finger domain (Kozaki et al., 2004). These findings are of great significance for understanding the genetic regulatory network of maize flowering time. However, maize flowering time is a typical quantitative trait and is jointly regulated by multiple genes (Buckler et al., 2009). Therefore, it is necessary to further analyse the genetic basis of flowering time regulation in maize and to explore new genetic regulatory loci.

Linkage analysis and genome-wide association studies (GWASs) have proven to be effective methods for mining quantitative trait loci (QTLs) for important traits, including maize flowering time. Buckler et al. (2009) systematically studied the genetic basis of maize flowering time by using the nested association mapping (NAM) population, which contains 5000 recombinant inbred lines (RILs). On this basis, an additive genetic model was proposed to explain the flowering time of maize: maize flowering time is controlled by multiple micro-QTLs (Buckler et al., 2009). In subsequent studies, Li et al. (2016) identified nearly 1000 significant single nucleotide polymorphisms (SNPs), which were associated with 220 candidate genes related to maize flowering time, by using an extensive association mapping population of more than 8000 lines. Notably, due to the high diversity among maize inbred lines, it is difficult to determine all the factors regulating flowering time (Hufford et al., 2021). Moreover, the significant loci identified for different populations are not consistent (Li et al., 2016). Therefore, it is necessary to investigate additional populations to discover new genetic regulatory loci and candidate genes for flowering time to provide a basis for subsequent maize germplasm improvement.

In this study, we used two maize germplasm panels, (i) a natural association panel with 226 inbred lines and (ii) a linkage mapping population with 120 F_2:3_ offspring obtained from the cross between the T32 and Qi319 maize lines, to identify the genetic loci associated with maize flowering time. Three flowering time-related traits (DTT, days to tasselling; DTP, days to pollen shed; DTS, days to silking) were analyzed in different environments. The GWAS approach, QTL analysis and transcriptomic analysis were combined in this study to identify new loci and reveal candidate genes for maize flowering time.

## 2 Materials and methods

### 2.1 Plant materials

A linkage population with 120 F_2:3_ offspring developed from parents of the maize T32 (renamed Ki32) and Qi319 lines and the association mapping panel with 226 inbred lines were selected as the plant experimental materials in this study. T32 is a tropical maize line derived from the Suwan population and is widely used for breeding in southern China. Qi319 is a temperate line and has been widely used in temperate regions, especially in northern China (Wu et al., 2019).

### 2.2 Field experiment and flowering time-related trait evaluation

The association panel materials were planted and phenotyped in Guiyang (GY, 106.7°N, 26.5°E), Guizhou Province; in Sanya (SA, 18.36°N, 109.16°E), Hainan Province; and in Zhangye (ZY, 38.93N, 100.45°E), Gansu Province in 2020. The linkage population was planted and phenotyped in Sanya (2019) and Guiyang (2020). Each line was planted in a single row 3 m in length with 12 individual plants per row. The fertilization, irrigation, pest control and weed management for all field trials were the same as those of the local field. Three flowering time-related traits (DTT, DTS, DTP) were recorded when 50% of plants exhibited the corresponding traits.

### 2.3 DNA extraction and genotyping

Genomic DNA was extracted from young leaves of F_2_ plants using the cetyltrimethylammonium bromide (CTAB) procedure based on our previously described methods (Wu et al., 2015). DNA quality testing and genotyping by sequencing (GBS) assessments were completed by the Beijing Compass Biotechnology Company by using previously described methods (Elshire et al., 2011). The high-quality SNPs between parents were identified by alignment with B73 RefGen_v4 using the BWA package and GATK (Lai et al., 2010; McKenna et al., 2010). The calling and annotation of SNPs were accomplished using SAMTOOLS software (Li and Durbin, 2009). In addition, genotyping, population structure detection, kinship determination, and principal component analysis (PCA) of the association panel had already been completed in our previous research (Wu et al., 2019).

### 2.4 QTL mapping and genome-wide association study

A total of 169,108 high-quality SNPs were selected to construct a genetic map using the ordering algorithm. QTL analyses were conducted using QTL IciMapping software Version 4.1 (Li et al., 2008). A total of 43,252 SNPs were selected to perform a phenotype–genotype GWAS by using TASSEL v5.2.80 software, with a mixed linear model (MLM) in which population structure and pairwise kinship were treated as covariates (Yu et al., 2006). The significant cut-off value was defined as a logarithm of odds (LOD) score >4.

### 2.5 Transcriptome data analysis

T32 and Qi319 were planted at the Sanya and Zhangye sites. Leaves were collected from three replicates of each inbred line at the V9 stage. A total of 42 samples were collected for total RNA extraction. Total RNA was collected by using TRIzol reagent, and the construction of cDNA libraries and RNA sequencing were performed by Biomarker Technologies (Beijing, China) with the Illumina HiSeq 2000 platform. The clean reads were mapped to the maize B73 reference genome assembly V4 by using TopHat2 (Trapnell et al., 2012). The gene expression level was estimated by using the fragments per kilobase per million reads (FPKM) value. The differentially expressed genes were obtained by using the R statistical software package DESeq with *P*_adj_ < 0.05 and | log_2_(fold change [FC])| ≥ 1 (Anders and Huber, 2010).

### 2.6 Candidate gene detection and qRT-PCR analysis

Based on the maize B73 reference genome assembly V4, genes located within two times the linkage disequilibrium distance of one quantitative trait nucleotide were determined to be candidate genes for flowering time-related traits. Functional annotations of these candidate genes were completed using the Protein–Protein Basic Local Alignment Search Tool (BlastP) and conserved domain search tools. The qRT-PCR primers were designed by Primer5 software and are listed in **Table S1**. The GAPDH gene was used for data normalization, and three biological replicates were used for each sample. To analyse the data, the 2^−(△△^ *^CT^* ^)^ method was used (Livak and Schmittgen, 2001).

## 3 Results

### 3.1 Phenotypic variation in different environments

**Figure 1:**
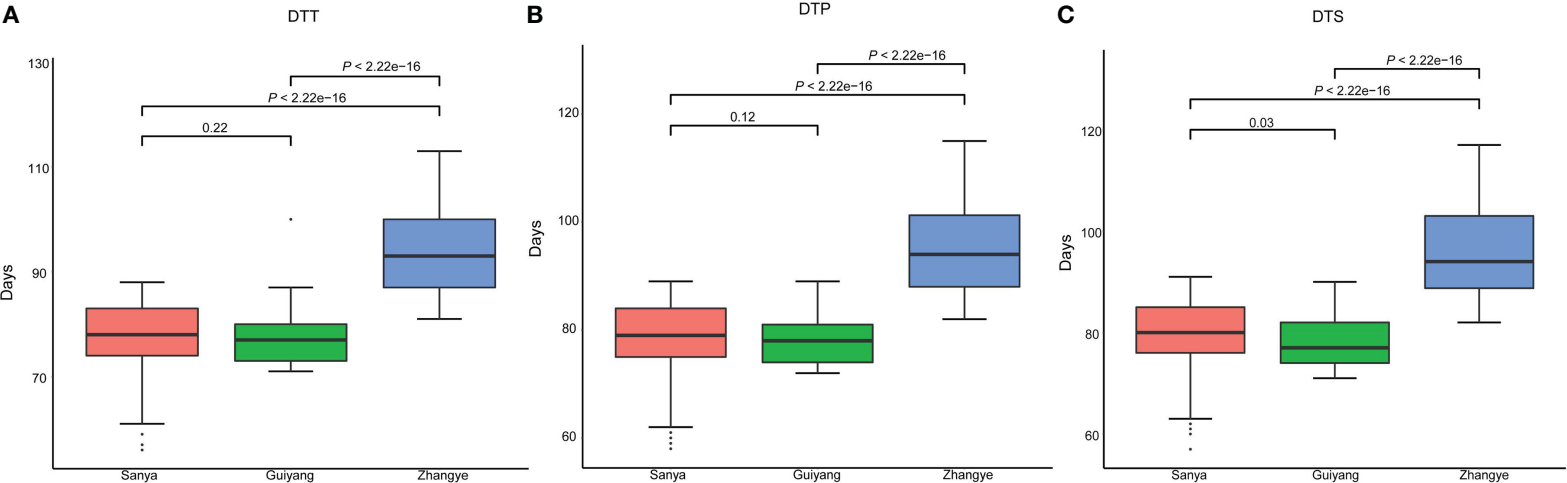
Flowering time-related traits for association mapping in three different site-specific environments (in Sanya, Guiyang and Zhangye). **(A)** days to tasselling (DTT); **(B)** days to pollen shed (DTP); **(C)** days to silking (DTS). A *t* test was used for analysis.

The results showed that the average DTT was 59.03 days in Sanya, 76.56 days in Guiyang, and 93.69 days in Zhangye (**Figure 1A**, **Table S2**). The average DTP was 60.11 days in Sanya, 77.57 days in Guiyang and 94.97 days in Zhangye (**Figure 1B**, **Table S2**). The average DTS was 60.91 days in Sanya, 77.98 days in Guiyang and 95.93 days in Zhangye (**Figure 1C**, **Table S2**). The population at the Zhangye site had a higher DTT, DTP and DTS than those at the Sanya and Guiyang sites (**Figure 1**). There were significant correlations between the three traits in different site-specific environments (**Table S3**). Significant effects of genotype and genotype × environment (*G×E*) were found for flowering time-related traits in the association panel (**Table S4**). The *H^2^* for DTT, DTP and DTS was 0.75, 0.74 and 0.74, respectively, as calculated as described in a previous study. These results showed that flowering time was influenced by the environment.

Based on our previous results, the association mapping panel could be divided into seven subgroups (HCL645 subgroup, T32 subgroup, QR273 subgroup, Mo17 subgroup, A801 subgroup, B73 subgroup and mixed subgroup). Based on the statistical analysis, population structure was not significantly different for the three traits (**Table S5**). Among the seven subgroups, the A801 subgroup had the greatest values for DTT, DTP and DTS, and the HCL645 subgroup had the smallest value for maize flowering time.

### 3.2 GWAS of maize flowering time

**Figure 2:**
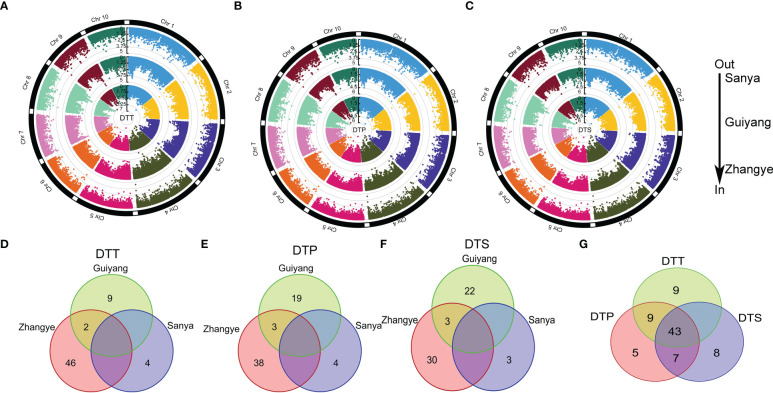
The GWAS for maize flowering time. **(A)** DTT; **(B)** DTP; **(C)** DTS. The three circles from outer to inner indicate three different local environments (in Sanya, Guiyang and Zhangye). **(D–F)** show the common significant SNPs in the three local environments for DTT, DTP and DTS, respectively. **(G)** shows that the common significant SNPs were simultaneously related to DTT, DTP and DTS.

To identify the significant loci (LOD >4) associated with maize flowering time, an MLM analysis was performed on the association panel (**Figure 2**, **Table S6**). For DTT, 48, 11 and 4 significant loci were found in the populations at the Zhangye, Guiyang and Sanya sites, respectively (**Figure 2A**, **Table S6**). For DTP, a total of 41, 22, and 4 significant loci were identified at the Zhangye, Guiyang and Sanya sites, respectively (**Figure 2B**, **Table S6**). Thirty-three, 25 and 3 loci were found to be significantly associated with DTS at the Zhangye, Guiyang and Sanya sites, respectively (**Figure 2C**, **Table S6**). The amount of phenotypic variation explained by these significant loci ranged from 9.2% to 14.0% (**Table S6**). PZE-106004147, which was found to be associated with DTT at the Sanya site and was located on Chr6, explained the least phenotypic variation, and PZE-108068611, which was found to be associated with DTS at Guiyang and located on Chr8, explained the most.

In addition, these significant loci related to maize flowering time were detected only in certain site-specific environments. In the three different site-specific environments (in Zhangye, Guiyang and Sanya), DTT, DTP and DTS were significantly correlated with only 2 (PZE-104072142 and PZE-104096936), 3 (PZE-101256915, PZE-104072142 and PZE-104096936) and 3 (PZE-108090522, PZE-104072142 and PZE-104096936) loci, respectively (**Figures 2D–F**). Here, a total of 43 loci were found to be significantly associated with DTT, DTP and DTS simultaneously (**Figure 2G**). There were also 9 loci that regulated DTT and DTP simultaneously and 7 loci that regulated DTP and DTS simultaneously. These results indicated that there is a significant genetic correlation between flowering time-related traits and that these traits are highly susceptible to site-specific environmental impacts.

**Figure 3:**
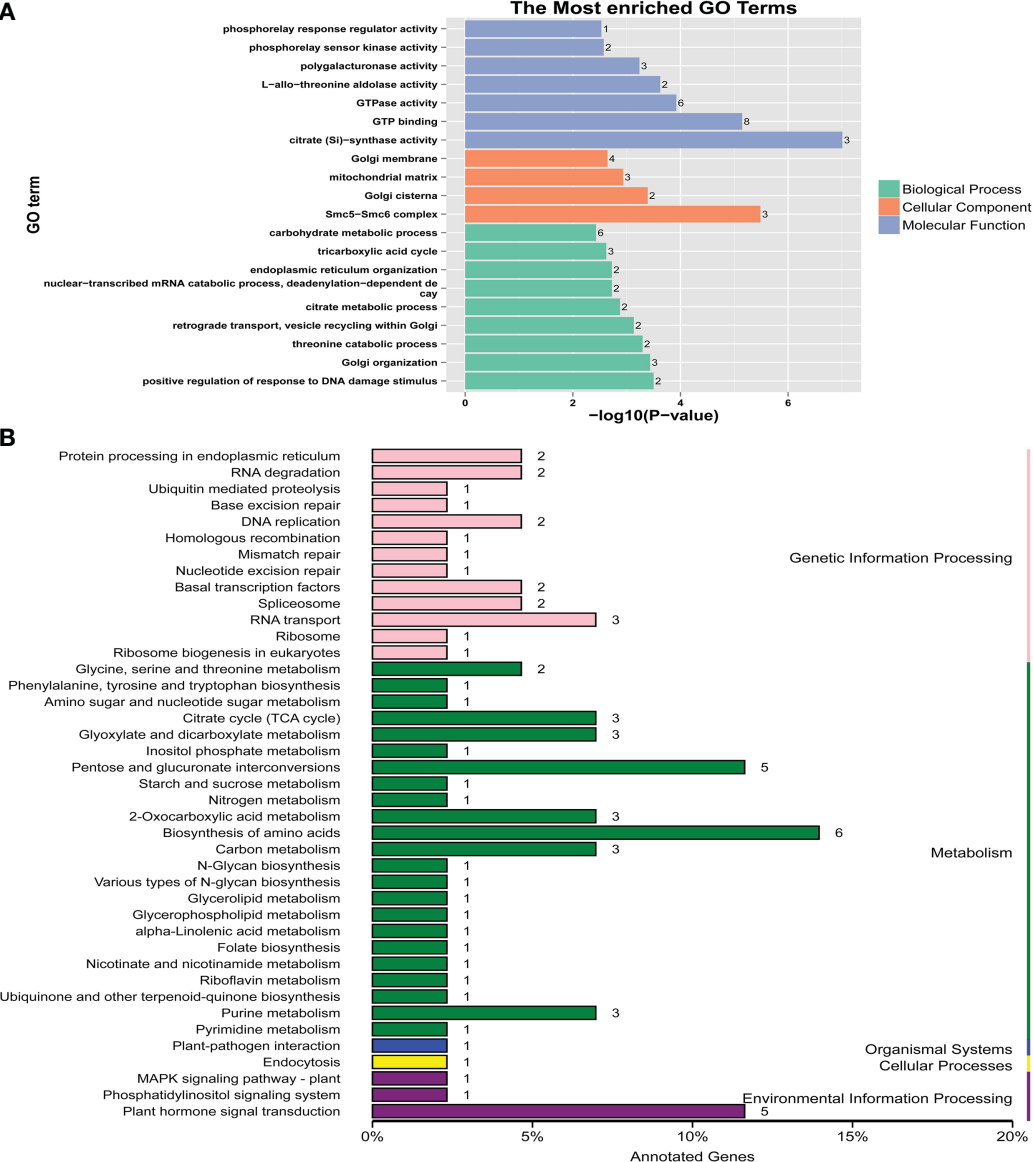
GO and KEGG analyses for the candidate genes identified by GWAS. **(A)** GO analysis. **(B)** KEGG analysis.

A total of 117 candidate genes were found around the 82 significant SNPs (**Table S6**). Among these candidate genes, some genes known to be related to flowering time in plants were detected. For example, Zm00001d050018 (*bzip68*) encodes the ABI5 protein, and its homologue in Arabidopsis delays flowering (Chang et al., 2019). Zm00001d044272 (*bhlh94*) encodes the bHLH transcription factor, and homologous genes can upregulate the expression level of *FT* to regulate flowering time in Arabidopsis (Li et al., 2017). Gene Ontology (GO) analysis and Kyoto Encyclopedia of Genes and Genomes (KEGG) analysis were conducted for the 117 candidate genes. The GO results showed that eight genes were involved in GTP binding and six genes were involved in the carbohydrate metabolic process (**Figure 3A**). The KEGG results showed that these genes were involved in four main processes (genetic information process, metabolism, organismal systems cellular process and environmental information process), six genes were involved in the amino acid biosynthesis process, and five genes were involved in the plant hormone signal transduction process (**Figure 3B**).

### 3.3 QTL analysis

**Figure 4:**
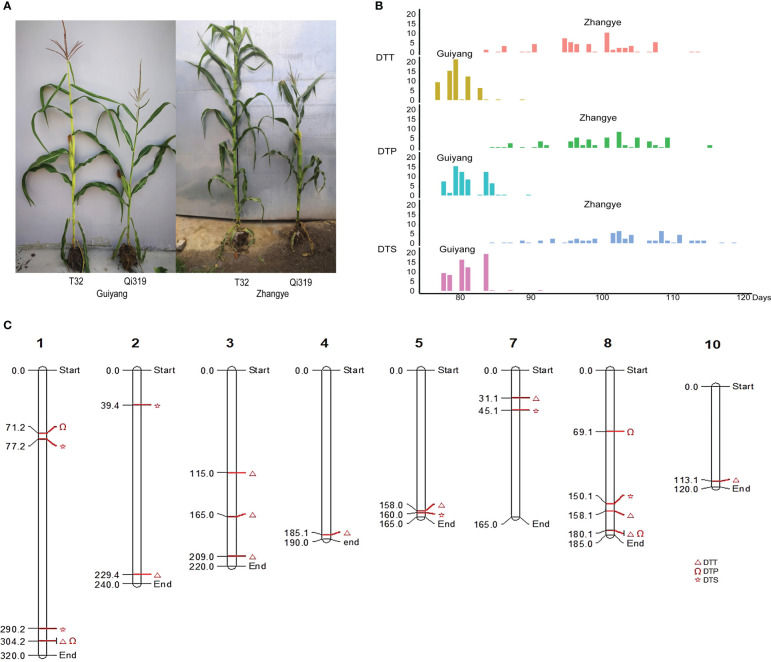
Linkage analysis for flowering time in the F_2:3_ population. **(A)** Performance of the parents (T32 and Qi319) in the different site-specific environments (Guiyang and Zhangye). **(B)** The distribution of DTT, DTP and DTS in the F_2:3_ population at the Zhangye site and in Guiyang. **(C)** The QTLs identified in this population.

T32 and Qi319 exhibited significant differences in flowering time in different environments (**Figure 4A**). T32 had a longer flowering time than Qi319 in Guiyang and Zhangye (**Table S7**). For DTT, DTP and DTS, this F_2:3_ population showed more variation at the Zhangye site than at the Guiyang site (**Figure 4B**, **Table S7**). In this population, the *H^2^* values for DTT, DTP and DTS were 0.559, 0.557 and 0.558, respectively, which suggests that the environment plays an important role in maize flowering time. A total of 169,108 SNP markers were used to construct the genetic linkage map (**Figure S1**). The SNP number for each chromosome ranged from 12,112 (Chr9) to 23,145 (Chr1).

For DTT, a total of eleven QTLs were found in the Zhangye (eight QTLs) and Guiyang (three QTLs) environments (**Figure 4C**, **Table S8**). The phenotypic variation in DTT explained by each locus ranged from 11.4% (*qZYDTT5*) to 32.5% (*qGYDTT1*). For DTP, four QTLs (one QTL found at the Zhangye site and three QTLs found in Guiyang) were identified in this population (**Figure 4C**, **Table S8**). The amount of phenotypic variation explained ranged from 22.4% (*qZYDTP1*) to 35.4% (*qGYDTP2*). For DTS, six QTLs were found in the Zhangye (three QTLs) and Guiyang (three QTLs) environments (**Figure 4C**, **Table S8**). The phenotypic variation ranged from 7.8% (*qZYDTS2*) to 32.6% (*qGYDTS1*). Among the QTLs related to DTT, DTP and DTS, two genetic regions exhibited pleiotropic effects: *qGYDTT1* and *qGYDTP1* were both located at 304.2 Mb on Chr1, and *qGYDTT3* and *qGYDTP3* were both located at 180.1 Mb on Chr8 (**Table S8**). These results suggested that these two genomic regions can simultaneously regulate DTT and DTP in maize.

Sixty-five candidate genes were detected in these QTL interval regions, and some important genes related to plant flowering time were found. For example, Zm00001d011669, which is located in the *qZYDTT7* region, encodes an MYB transcription factor; Zm00001d029584, which is located in the *qZYDTS1* region, encodes a zinc finger protein; and Zm00001d003293 encodes an NAC transcription factor.

### 3.4 Transcriptome analysis

**Figure 5:**
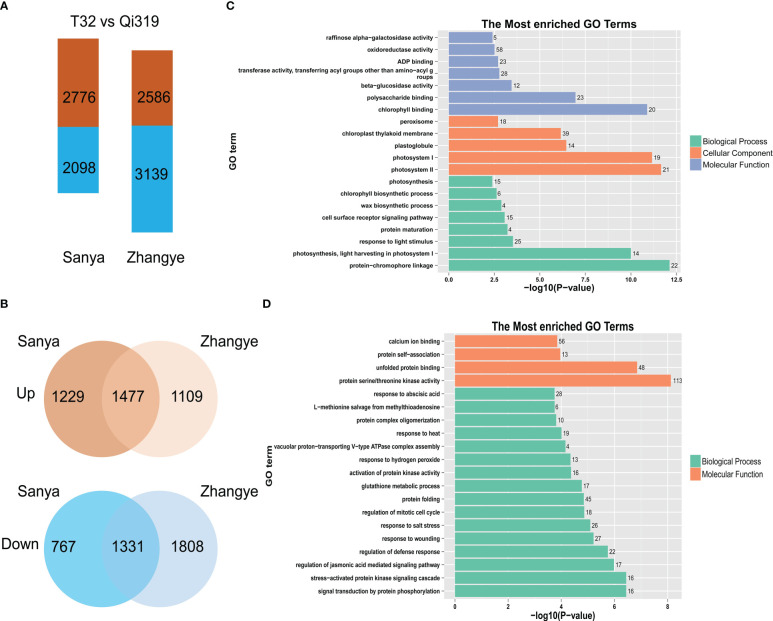
The DEGs identified between T32 and Qi319 in different site-specific environments (in Zhangye and Sanya). **(A)** The number of DEGs in different environments. **(B)** The common DEGs in different environments. **(C)** The most enriched GO terms for the upregulated genes. **(D)** The most enriched GO terms for the downregulated genes.

To identify the genes involved in maize flowering time, the differentially expressed genes (DEGs) between T32 and Qi319 in different environments (Sanya and Zhangye) were identified (**Figure 5A**, **Tables S9**, **S10**). In Sanya, 2776 genes were upregulated and 2098 genes were downregulated in Qi319 compared with T32. In Zhangye, 2586 genes were upregulated and 3139 genes were downregulated in Qi319. Among these DEGs, 1477 common genes were upregulated and 1331 common genes were downregulated in the two different environments (**Figure 5B**). To identify the biological functions of these DEGs, GO enrichment analyses were conducted. There was a significant difference among the upregulated and downregulated genes. Among the upregulated genes, 58 genes were involved in oxidoreductase activity, 25 genes were related to light stimulus, 23 genes were involved in polysaccharide binding, and 15 genes were involved in photosynthesis (**Figure 5C**). Among the downregulated genes, 113 genes had protein serine/threonine kinase activity, 45 genes were involved in the protein folding process, and 28 genes were involved in the ABA process (**Figure 5D**).

### 3.5 Combining linkage analysis, the GWAS approach and transcriptome analysis to identify candidate genes for maize flowering time

**Figure 6:**
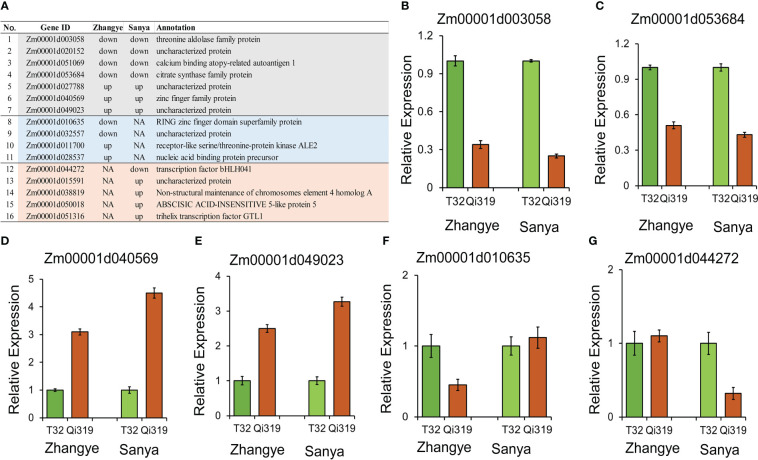
Candidate genes identified by GWAS and transcriptome analysis. **(A)** Information on sixteen DEGs. B-G, qRT-PCR results for selected genes. **(B)** Zm00001d003058; **(C)** Zm00001d053684; **(D)** Zm00001d040569; **(E)** Zm00001d049023; **(F)** Zm00001d010635; **(G)** Zm00001d044272.

To identify the candidate genes for maize flowering time, the results of GWASs and linkage and transcriptome analyses were integrated. Among the 117 candidate genes identified by using GWAS, 16 genes were differentially expressed between T32 and Qi319 (**Figure 6A**). Among these 16 genes, six genes were differentially expressed in both environments. For example, the expression levels of Zm00001d003058, which encodes a threonine aldolase protein (**Figure 6B**), and Zm00001d053684, which encodes a citrate synthase protein, were higher in T32 than in Qi319 in Zhangye and Sanya (**Figure 6C**). The expression levels of Zm00001d040569 and Zm00001d049023 were higher in Qi319 than in T32 in the two environments (**Figures 6D, E**). In addition, we also found that four genes had different expression levels only in Zhangye. For example, Zm00001d010635, which encodes a zinc finger protein, differed in expression between T32 and Qi319 only in Zhangye (**Figure 6F**). Five genes were found to have different expression levels in Sanya, such as Zm00001d044272, which encodes a bHLH transcription factor (**Figure 6G**).

**Figure 7:**
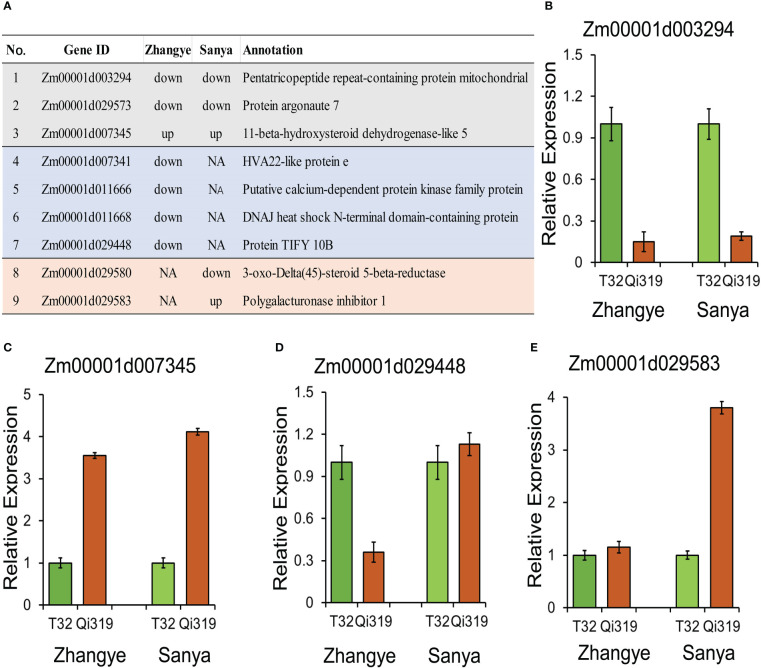
Candidate genes identified by QTL mapping and transcriptome analysis. **(A)** Information on nine DEGs. **(B–E)** qRT-PCR results for selected genes. **(B)** Zm00001d003294; **(C)** Zm00001d007345; **(D)** Zm00001d029448; **(E)** Zm00001d029583.

Among the 65 candidate genes identified by QTL analysis, 9 genes were differentially expressed between T32 and Qi319 (**Figure 7A**). Three genes were differentially expressed in the two environments: Zm00001d003294 was downregulated in Qi319 at the Zhangye and Sanya sites (**Figure 7B**), and Zm00001d007345 was upregulated in Qi319 at the Zhangye and Sanya sites (**Figure 7C**). Four genes were downregulated in Qi319 only at the Zhangye site, such as Zm00001d029448, which encodes a TIFY 10B protein (**Figure 7D**). Two genes were differentially expressed only in Sanya. The results of qRT-PCR were consistent with the results of transcriptome analysis (**Figures 7B–E**).

**Figure 8:**
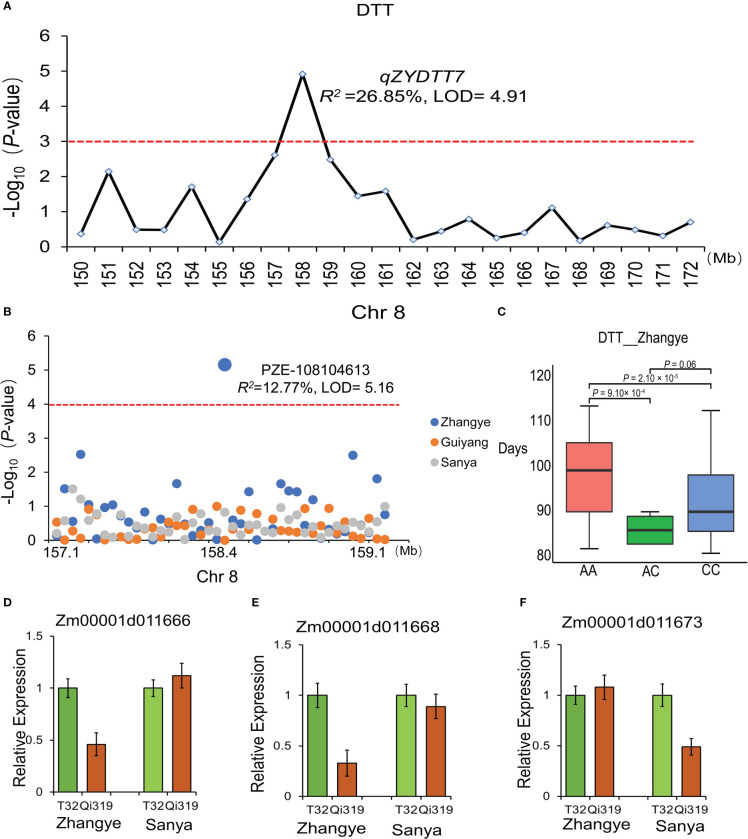
The candidate genes identified by the combination of QTL analysis, the GWAS approach and transcriptome analysis. **(A)** *qZYDTT7*, which was found to be significantly related to DTT at the Zhangye site, is located on Chr8 (158 Mb). **(B)** PZE-105104613, which was found to be related to DTT at the Zhangye site and is located in the same genomic region. **(C)** Haplotype analysis of PZE-105104613 with DTT. T tests were used for analysis. **(D–F)** The expression of the three candidate genes in the T32 and Qi319 lines in different site-specific environments. **(D)** Zm00001d011666; **(E)** Zm00001d011668; **(F)** Zm00001d011673.

To further narrow the genomic regions related to maize flowering time, the results of QTL analysis and the GWAS approach were integrated in this study. Interestingly, one major QTL related to DTT, which was found in plants at the Zhangye site (*qZYDTT7*, LOD = 4.91, *R^2 =^* 26.85%), was identified by GWAS (**Figures 8A, B**). PZE-108104613, which is located in the interval of *qZYDTT7*, was significantly (LOD = 5.16, *R^2 =^* 12.77%) related to DTT in plants at the Zhangye site. This SNP has two alleles (A and G), and the average DTT associated with the A allele (97.8 days) was significantly different from that associated with the G allele (92.1 days) (**Figure 8C**). Based on the B73 reference genome, a total of 17 genes were found in this interval (Chr8, 158-159 Mb). Among these genes, Zm00001d011666, which encodes a calcium-dependent protein kinase family protein, and Zm00001d011668, which encodes the DNAJ family protein, had different expression levels between T32 and Qi319 in Zhangye (**Figure 8D, E**), and Zm00001d011673, which is also named *fps2* and is related to the development of maize leaves, had different expression level between T32 and Qi319 at the Sanya site (**Figure 8F**).

## 4 Discussion

Compared with teosinte, which can only grow in tropical environments, maize has become one of the most widely planted crops in the world (Yang and Yan, 2021). The main reason is that maize adapts to different geographical environments *via* flowering time regulation (Li et al., 2016). During Chinese maize breeding practices, T32 was a foundation parental line derived from the Suwan germplasm, which showed a high combining ability but a longer reproductive period. It matured later than another foundation parental line, Qi319, derived from temperate maize germplasm in tropical regions. T32 cannot flower normally in temperate environments based on breeding experience, but Q319 can flower normally in different environments. Therefore, maize flowering time is an important characteristic that determines local environmental adaptation and is easily affected by the local environment. Similar to the findings of a previous study (Shi et al., 2022), the interaction effect of genotype and the environment on maize flowering time-related traits was significant in this study. Revealing the potential genetic basis of flowering time will aid in the selection of stable varieties in different local environments and will improve maize yields.

The GWAS approach has been shown to be an effective strategy for mining genetic loci for flowering time in maize (Buckler et al., 2009; Li et al., 2016; Shi et al., 2022). For example, a total of 18 SNPs and 19 candidate genes involved in maize flowering time were found in an association panel that consisted of 252 inbred lines (Vanous et al., 2018), and Li et al. (2016) identified nearly 1000 SNPs and 220 candidate genes using an extremely large association panel. In this study, a total of 82 SNPs and 117 candidate genes for maize flowering time-related traits were found in plants growing in different site-specific environments. Compared with the results of Li et al. (2016), 14 common SNPs were found in our study (**Table S6**). Among these candidate genes, some important candidate genes for flowering time were found. For example, Zm00001d007191 encodes an MYB transcription factor, and many previous studies have shown that MYB transcription factors, such as *MYB30* (Liu et al., 2014), *MYB106* (Hong et al., 2021), and *CmMYB2* (Zhu et al., 2020), play an important role in the development of flowers. Zm00001d044272 encodes a *bHLH* transcription factor. In Arabidopsis, the *bHLH* transcription factors *MYC*2, *MYC3*, and *MYC4* delay flowering time *via* the jasmonate pathway (Wang et al., 2017). In rice, two bHLH transcription factors (HBP1 and POH1) control flowering time by regulating the expression level of *Hd1* (Yin et al., 2023). In addition, Zm00001d050018, which encodes the ABI5 protein, was also found to be an important candidate gene for maize flowering time. In Arabidopsis, AtU2AF65b functions in abscisic acid (ABA)-mediated flowering by regulating the precursor messenger RNA splicing of ABI5 (Xiong et al., 2019).

QTL analysis is another effective method for mining the genetic loci for quantitative traits (Maschietto et al., 2017; Su et al., 2017; Wang et al., 2018). The resolution of QTL analysis can be enhanced by using a high-density genetic map (Maschietto et al., 2017; Su et al., 2017; Wang et al., 2018). In this study, a high-density genetic map was constructed with 169,108 markers by using the GBS method. In this study, we found nine QTL regions that were also found in a previous study (**Table S8**). For example, *qZYDTT1* and *qZYDTT3* were also found in Li et al. (2016) as *CN_QTL_17* and *CN_QTL_21*, respectively. These results showed that the nine QTL regions may be hotspot regions related to maize flowering time. Among the 65 candidate genes, some genes have been identified as important for plant flowering time. For example, Zm00001d023424 encodes the bZIP transcription factor, and Zm00001d047250 encodes the PLATZ transcription factor. In rice, *OsbZIP62*, which is a functional orthologue of FLOWERING LOCUS D, regulates the floral transition and panicle development (Kaur et al., 2021). In grapevine, *VviPLATZ1* is a major factor that controls female flower morphology determination (Iocco-Corena et al., 2021).

The combination of the GWAS approach, linkage analysis and transcriptome analysis can help us quickly identify candidate genes. For example, *ZmWRKY14*, which is a regulator of maize leaf number, flowering time and biomass yield, has been identified based on GWAS and linkage analysis (Li et al., 2021). By using the same strategy, seventeen candidate genes significantly associated with maize flowering time and leaf number have been found (Shi et al., 2022). In this study, 25 important candidate genes were found by integrating the results of GWASs, QTL mapping, and transcriptome analysis. Among the 25 DEGs, some genes had an important role in plant flowering time, such as Zm00001d044272, which encodes the bHLH transcription factor, and Zm00001d05008, which is the ABI5 gene. In addition, we found that one genome region located on Chr 8 (158 Mb) was significantly associated with DTT, which we were able to identify simultaneously by linkage analysis and GWAS. After combining these results with the results of transcriptome analysis, three important candidate genes were found. Zm00001d011666 encodes a calcium-dependent protein kinase (CPK) family protein. A previous study showed that the CPK32 gene can control pollen tube growth in tobacco and maize (Zhou et al., 2014; Li et al., 2018). Zm00001d011668 encodes the DNAJ family protein, and previous studies have shown that the DNAJ family protein plays an important role in seed filling and abiotic stress response (Hajduch et al., 2010). Zm00001d011673, which is also named *fps2*, encodes the farnesyl diphosphate synthase 2 protein. A previous study showed that Zm00001d011673 can interact with ZmIPT2, which can regulate leaf senescence and grain yield in maize (Song et al., 2022).

In conclusion, in this study, a total of 82 significant SNPs with 117 candidate genes and 21 QTLs with 65 candidate genes associated with maize flowering time were found by using the GWAS approach and QTL analysis, which can be evaluated and used in molecular-assisted breeding practices in the future. By combining the GWAS, QTL and transcriptome analysis results, 25 DEGs were found, and among these genes, three important candidate genes (Zm00001d011666, Zm00001d011668 and Zm00001d011673) were inferred as regulators of flowering time in maize. Our results provide an important gene resource for maize breeding to improve flowering time.

## Data availability statement

The datasets presented in this study can be found in online repositories. The names of the repository/repositories and accession number(s) can be found in the article/**Supplementary Material**.

## Author contributions

XW: Conception and design, Conceptualization, Methodology, Validation, Formal analysis, Data curation, Writing - original draft. YL: Contributed to some of the experiments, Formal analysis, Data curation. XL: Conceptualization, Formal analysis, Validation, Data curation. LT: Contributed to some of the experiments. YG: Contributed to some of the experiments. DW: Contributed to some of the experiments. SG: Contributed to some of the experiments. YX: Contributed to some of the experiments. PX: Contributed to some of the experiments. XG: Contributed to some of the experiments. AW: Contributed to some of the experiments. PL: Contributed to some of the experiments. YZ: Contributed to some of the experiments. LC: Conception and design, Conceptualization, Methodology, Validation, Data curation, Writing - review & editing. ZC: Funding acquisition, Supervision, Methodology, Project administration. All authors contributed to the article and approved the submitted version.
