# Supplementary figures and images for: Integration of GWAS, linkage analysis and transcriptome analysis to reveal the genetic basis of flowering time-related traits in maize
Source: Front Plant Sci. 2023 Mar 22;14:1145327. doi: 10.3389/fpls.2023.1145327 (PMC10073556; doi:10.3389/fpls.2023.1145327)

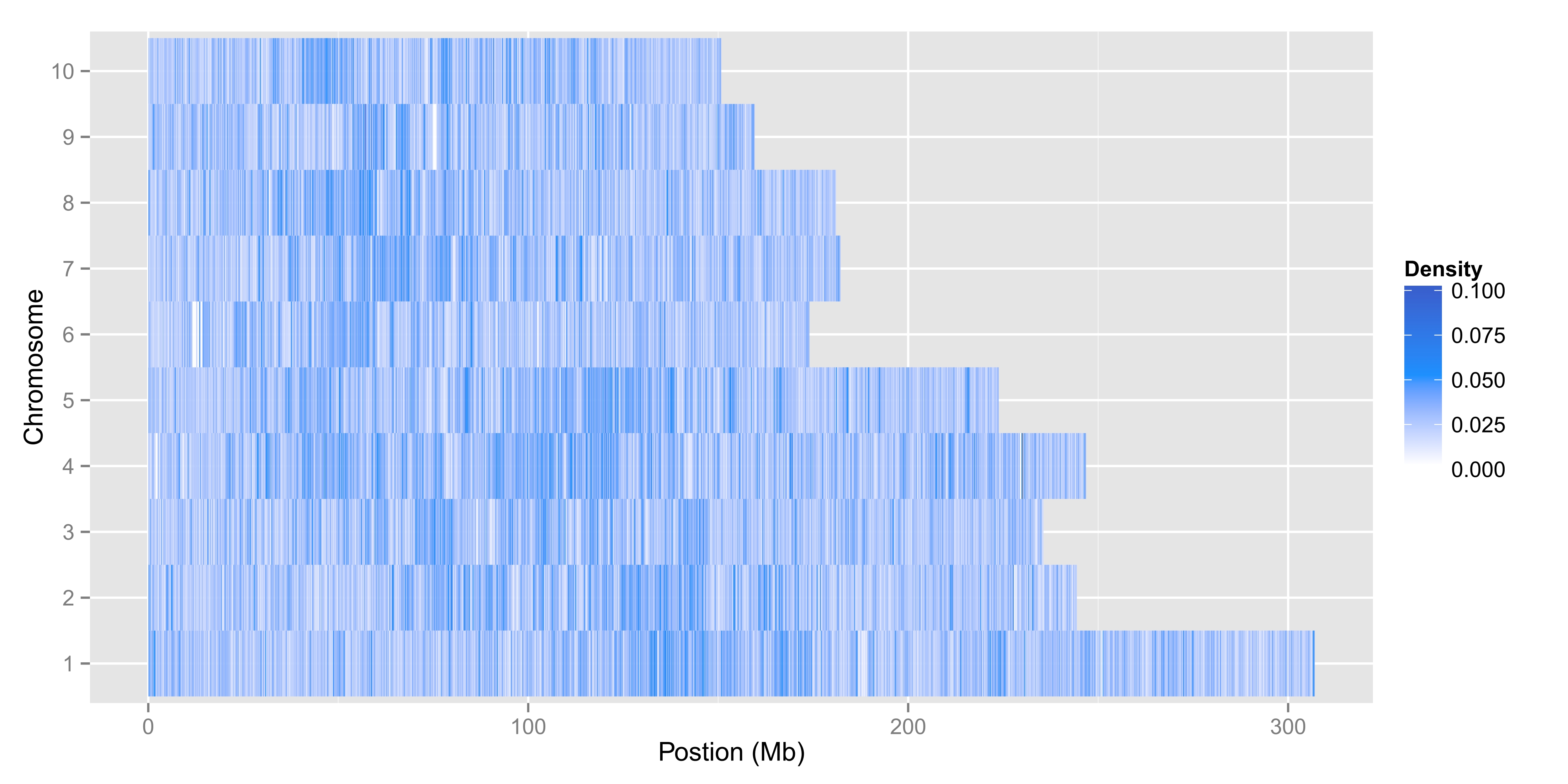

Supplement: Supplementary file 1 [file Image_1.jpeg]
